# Intimate Partner Violence and HIV Prevention Among Transgender and Nonbinary Persons: Protocol for a Prospective Mixed Methods Cohort Study

**DOI:** 10.2196/82090

**Published:** 2025-12-30

**Authors:** Erik D Storholm, Keith J Horvath, Adedotun Ogunbajo, Ayden Scheim, Arjee Restar, Ruby Lucas, Sarita D Lee, Sungsub Choo, Benji Jensen, Jessica Randazzo, Joshua Wolf, Cleo Spencer, Audren Bambilla, Glenn J Wagner

**Affiliations:** 1 School of Public Health San Diego State University San Diego, CA United States; 2 RAND Behavioral and Policy Sciences Santa Monica, CA United States; 3 Center for HIV Identification, Prevention and Treatment Services Department of Family Medicine University of California Los Angeles Los Angeles, CA United States; 4 Department of Psychology San Diego State University San Diego, CA United States; 5 Department of Epidemiology and Biostatistics Dornsife School of Public Health Drexel University Philadelphia, PA United States; 6 School of Public Health Yale University New Haven, CT United States; 7 Department of Epidemiology University of Washington Seattle, WA United States; 8 RAND Survey Research Group Santa Monica, CA United States

**Keywords:** intimate partner violence, IPV, transgender, health equity, HIV prevention, pre-exposure prophylaxis, PrEP

## Abstract

**Background:**

Transgender and nonbinary (TNB) individuals experience intimate partner violence (IPV) at twice the rates of cisgender populations. Although prior research has linked IPV to elevated HIV risk and vulnerability among TNB persons, there is limited understanding of how IPV influences key HIV prevention behaviors, such as HIV and sexually transmitted infection (STI) testing, and initiation and use of pre-exposure prophylaxis (PrEP). IPV experiences among TNB individuals are complex and diverse, varying by type, frequency, severity, and power and relationship dynamics, and often intersect with systemic forms of marginalization. Additional research is needed to investigate the mechanisms linking IPV and HIV outcomes to inform effective, tailored prevention strategies.

**Objective:**

This prospective mixed methods cohort study seeks to advance understanding of the risk and protective pathways between IPV (both perpetration and victimization) and HIV-related outcomes, including engaging in condomless sex, STI acquisition, PrEP uptake, adherence, and persistence among TNB individuals experiencing IPV.

**Methods:**

This study includes two sequential phases. Phase 1 consisted of formative qualitative interviews with 32 TNB individuals with recent IPV experience and 10 key informants (eg, service providers and advocates) in the United States. These interviews informed the design of a national, web-based cohort study. Phase 2 will enroll 600 HIV-negative, currently partnered TNB participants living in the United States. Participants will be followed for 24 months, with surveys and at-home biospecimen collection (HIV and STI testing and PrEP adherence) at baseline, 6, 12, 18, and 24 months. Brief surveys assessing changes in key variables will also be completed at 3, 9, 15, and 21 months.

**Results:**

Phase 1 was initiated in October 2023, with interviews conducted through October 2024 until thematic saturation was reached. Rapid qualitative analysis was completed between November 2024 and January 2025 to inform measurement selection for the phase 2 surveys. Enrollment for phase 2 began in February 2025 and is expected to continue through December 2025.

**Conclusions:**

This study will provide essential insights into how IPV impacts HIV risk and prevention practices among TNB individuals. Results will guide the development or refinement of gender-affirming, trauma-responsive, and culturally grounded IPV and HIV prevention interventions tailored to the needs of TNB communities.

**International Registered Report Identifier (IRRID):**

DERR1-10.2196/82090

## Introduction

Transgender and nonbinary (TNB) individuals experience intimate partner violence (IPV) at rates twice that of their cisgender peers [1-3]. Over half (54%) of TNB persons in the United States report having experienced some type of IPV, including acts involving coercive control or physical harm [4]. IPV is associated with condomless sex, sexually transmitted infection (STI), and HIV among TNB individuals [5-8]. A review of 88 studies found a high burden of HIV among transgender populations, with laboratory-confirmed prevalence estimates of 14.1% among transgender women and 3.2% among transgender men [9]. HIV prevalence was highest among Black transgender individuals (44.2%) [9]. The study also revealed significant prevention gaps, with 27% reporting no prior HIV testing and fewer than half aware of HIV pre-exposure prophylaxis (PrEP) [9]. Nonbinary individuals have been largely excluded from these estimates and continue to be underrepresented in epidemiological and intervention research.

The disproportionately high HIV risk among TNB individuals is largely shaped by structural factors—including stigma, discrimination, and systemic exclusion from health, legal, and economic institutions [9-13]. Barriers such as limited access to gender-affirming care, economic marginalization, and medical mistrust reduce engagement in prevention and treatment [14-17]. Many TNB individuals face compounding mental health challenges, and some may engage in survival economies, including sex work, which further increases their vulnerability to HIV [14,18,19]. These intersecting factors also contribute to heightened vulnerability to IPV [2,20,21], which itself may disrupt engagement in the HIV prevention continuum (HPC)—including HIV and STI testing, PrEP initiation, adherence, and persistence—by inducing fear, controlling behavior, and limiting autonomy in health decision-making [22,23].

Despite growing recognition of these disparities, the current evidence base on IPV among TNB populations remains limited in scope, quality, and specificity. For example, the impact of IPV on HPC outcomes may vary among TNB subgroups (ie, transmasculine, transfeminine, and nonbinary persons) and be influenced by other contextual factors such as gender expression, stage of transition, partner dynamics, and relationship type. Methodological limitations have constrained the state of knowledge regarding IPV among TNB subgroups. The limited prior work has been cross-sectional with diverse recall periods, greatly limiting causal and temporal inferences about the mechanisms underlying the associations found between IPV and HPC outcomes [2,20]. Combining TNB individuals with other populations, such as cisgender sexual minority men, has obscured the study of subgroup-specific dynamics and experiences of violence (ie, TNB-specific, psychological, emotional, sexual, and physical), frequency, escalation, and directionality of IPV within the relationships of TNB persons [2]. Additionally, most prior studies used measures of IPV that were developed for cisgender heterosexual populations and may fail to capture forms of abuse specific to TNB individuals, such as partner interference with gender affirmation or threats to disclose gender identity without consent [6-8,24]. The field also lacks data on key structural and interpersonal drivers of IPV among TNB individuals (eg, early life trauma, housing instability, social and community isolation, partner characteristics, and gender role ideologies) and how these shape HPC outcomes [6-8,24]. These gaps in our current understanding of IPV in TNB communities highlight the need for more rigorous research approaches to better explain these relationships.

Importantly, while IPV victimization has received some attention in the literature, IPV perpetration among TNB individuals remains understudied [2]. Very few studies have examined bidirectional IPV, or how violence manifests and is experienced across different relationship types, partner genders, or sexual orientations [2]. There has also been little differentiation between acts of self-defense and intentional perpetration, or between the genders of the individuals involved (ie, transmasculine, transfeminine, and nonbinary persons and cisgender male or female partners) [1]. IPV-like behaviors, such as physical altercations, have not been analyzed with appropriate nuance to distinguish intent, context, self-defense, or power dynamics. Additionally, although studies have identified an association between IPV and HIV seroconversion risk among TNB individuals [5,6,25-27], the mechanisms underlying this relationship are not well understood, and few studies have examined how IPV influences HPC engagement specifically [14,15,22,23,28,29].

Emerging evidence suggests that IPV and general experiences of violence may act as significant barriers to PrEP uptake and persistence among TNB persons [14,22,30]. One recent study found that general violence victimization was negatively associated with PrEP use in TNB populations [31]. Our prior research similarly found that gender-based violence was associated with both failure to initiate PrEP and early discontinuation among TNB participants in a PrEP demonstration project [22]. Concerns about potential IPV triggered by conversations about HIV prevention have also been identified as barriers to PrEP adherence and disclosure in intimate relationships [28,31]. Yet the field lacks a comprehensive, longitudinal understanding of how IPV interacts with relational, social, and structural factors to influence trajectories of engagement in HIV prevention. Specific antecedents—such as undisclosed gender identity, gender affirmation dynamics, partner control, HIV serodiscordance, or threats to partner self-concept—may uniquely impact how IPV is experienced and how it impacts HPC engagement among TNB individuals.

To address these significant knowledge gaps, Project RADIANT (Relationships And Dynamics—Improving Advocacy for Nonbinary and Trans) was designed to examine how IPV influences HIV and STI risk and protective behaviors among TNB individuals and how it contributes to disparities in engagement across the HPC. This project will focus on three specific points of engagement in the HPC—(1) HIV and STI testing (awareness), (2) PrEP initiation (uptake), and (3) PrEP persistence (adherence and retention) [32]—and will examine how HPC engagement varies by TNB subgroup. The project also aims to advance the field methodologically by using a validated, TNB-specific IPV scale developed by Peitzmeier and colleagues [3,33,34], which captures TNB-specific experiences of both victimization and perpetration, such as partner interference with gender affirmation or threats of outing. These items will be combined with additional constructs derived from phase 1 qualitative interviews to offer a multidimensional understanding of the relationship between IPV and HPC engagement. The study will also consider possible confounding, mediating, and moderating variables—including resilience factors and community support—that may shape these outcomes over time.

**Figure 1:**
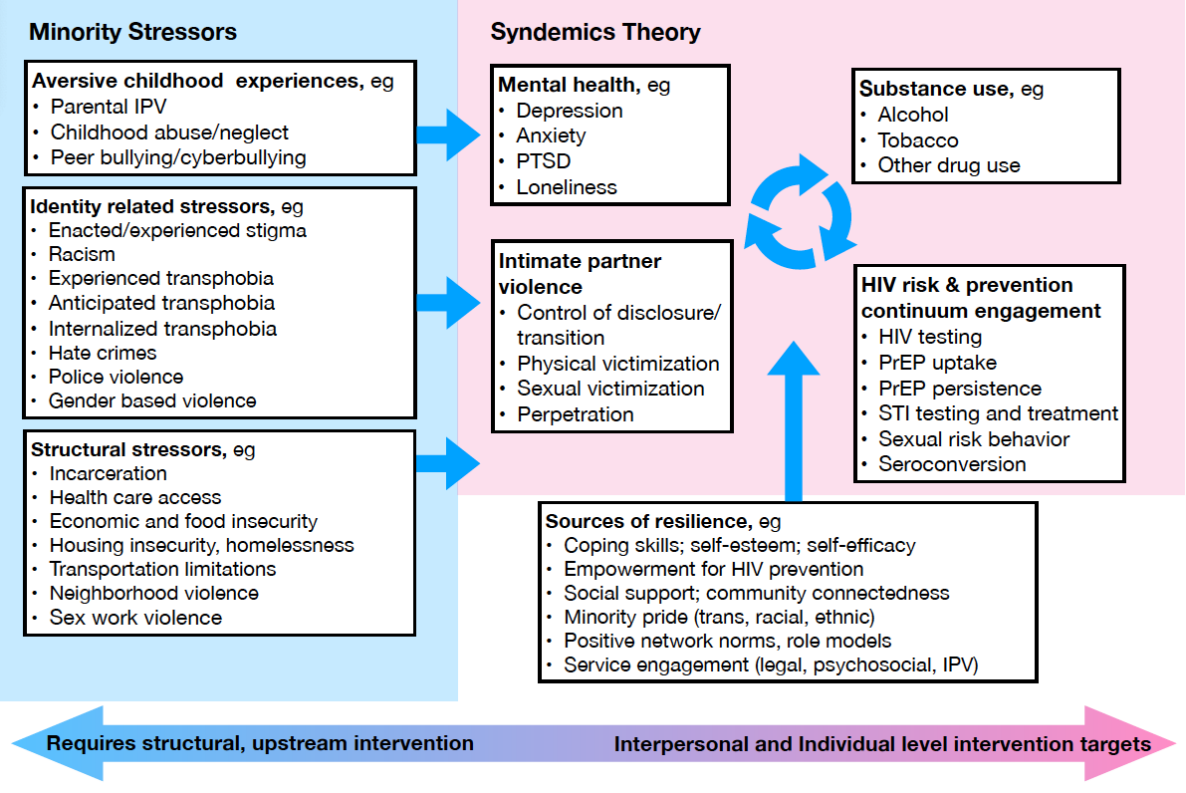
Conceptual model for IPV and HPC engagement among transgender and nonbinary persons. HPC: HIV prevention continuum; IPV: intimate partner violence; PrEP: pre-exposure prophylaxis; PTSD: posttraumatic stress disorder; STI: sexually transmitted infection.

This mixed methods, observational cohort study is guided by both syndemics theory [8,35-37] and the gender minority stress and resilience framework [38-44], which together provide a lens for understanding how IPV may influence engagement in the HPC among TNB individuals. Syndemics theory emphasizes how co-occurring psychosocial and health conditions, such as depression, substance use, trauma, and IPV, interact synergistically to worsen health outcomes like HIV, especially when shaped by shared social contexts [35,45,46]. These conditions do not arise in isolation, but are driven by upstream structural factors such as transphobia, racism, economic marginalization, and discriminatory legislation, which increase vulnerability to multiple, mutually reinforcing health challenges [35,45,46]. While these structural drivers (eg, legislation, stigma, racism, social exclusion, homelessness, poverty, and criminalization) are not themselves syndemic conditions, they create the environments in which syndemic conditions emerge and intensify risks among TNB persons [8,36,37,47,48]. The gender minority stress and resilience framework builds on this by focusing on the unique stressors experienced by TNB individuals due to their minoritized gender identity (eg, anticipated rejection, internalized stigma, and identity concealment) contribute to greater stress and poorer overall health while also recognizing the protective role of resilience factors like social support and community connectedness [36,49-51]. Both frameworks have been frequently applied to explore the underlying drivers of HIV inequities among TNB individuals, particularly in relation to the ways health disparities interact and amplify one another [8,36,37,47,48]. Together, these theories offer us guiding frameworks (Figure 1) for analyzing the broader consequences of IPV, extending beyond physical harm, to illuminate how IPV may directly and indirectly influence engagement in the HPC among TNB individuals, while also identifying possible points for intervention.

Anchored in these frameworks, this study seeks to address three major gaps in the literature: (1) the lack of longitudinal data linking IPV to HPC engagement among TNB individuals; (2) the widespread use of IPV measures developed for cisgender populations, which fail to capture TNB-specific experiences of abuse; and (3) the limited understanding of how subgroup differences (eg, transfeminine, transmasculine, and nonbinary) and relationship dynamics (eg, partner gender, power imbalances, and disclosure status) shape these associations. To fill these gaps, Project RADIANT will use a rigorous longitudinal cohort design, a validated TNB-specific IPV scale, along with additional items informed by phase 1 qualitative data, to assess a broad range of IPV experiences, including perpetration, directionality, coercive control, and interference with gender affirmation. By integrating these novel measurement tools into a longitudinal, community-informed study design, Project RADIANT aims to illuminate the pathways through which IPV affects HPC engagement and ultimately inform the development of effective, trauma-informed, and culturally responsive IPV and HIV prevention interventions to improve health outcomes for TNB communities.

## Methods

### Study Design

RADIANT is a prospective mixed methods cohort study. The study team comprises researchers and staff at San Diego State University, Drexel University, Yale University, the University of Washington, and RAND, with the RAND Survey Research Group programming and administering the survey components of the study. The study is being carried out in two phases. Phase 1 involved semistructured interviews with TNB persons with experiences of IPV and key informants, such as TNB-focused health care and social service providers working with TNB persons who have experienced IPV. The main purpose of phase 1 was to inform the selection of survey measures and activities for phase 2. Phase 2 is currently ongoing and involves the recruitment and retention of a prospective cohort of 600 TNB persons from across the United States who will complete online surveys and HIV and STI at-home test kits to assess subgroup differences in IPV and HPC engagement over 24 months.

### Community Advisory Board

The RADIANT study established a community advisory board (CAB) composed of TNB leaders and advocates to ensure community-centered research practices throughout the study. Initially, the project team consulted with subject matter experts, including members of existing TNB-specific CABs. Following these preliminary meetings, recruitment for the ongoing RADIANT CAB was conducted nationally through word-of-mouth and online advertisements via Instagram and Facebook. From these recruitment efforts, 224 individuals completed the interest screener, and 15 were selected to form the RADIANT CAB. Members were chosen to ensure representation across geography, age, gender identity, and racial or ethnic background, reflecting the diverse communities most affected by both IPV and HIV disparities within TNB populations.

The first CAB meeting took place in August 2024, and 8 additional meetings have been held to date. These ongoing virtual meetings ensure consistent engagement with the CAB and provide continual opportunities to incorporate their guidance on study design decisions (eg, study measurements and recruitment methods), methodological approaches, and interpretation of preliminary findings. Specifically, the CAB has provided critical input into the development of the study name, logo, and recruitment materials and methods, and has offered essential feedback on phase 1 interview questions, phase 2 survey development, and a conference presentation of phase 1 preliminary findings. CAB members are compensated for each meeting they attend and for their time spent providing in-depth feedback. This equitable, collaborative structure ensures that the research remains grounded in community knowledge and priorities, in service of TNB communities’ well-being [52]. CAB members have also opted to serve as coauthors on papers currently in development and to provide essential oversight to maintain the study's cultural responsiveness, trauma-informed practices, and overall relevance to TNB communities.

### Phase 1 Qualitative Data Collection

Formative qualitative interviews were conducted in phase 1 with a racially, ethnically, and gender-diverse sample of TNB persons who reported prior experiences of IPV (victimization or perpetration) within the past 12 months (n=32), as well as with key informants who provide services to TNB individuals experiencing IPV (n=10). This qualitative data collection aimed to explore relationship characteristics and dynamics, IPV experiences, IPV service usage, and HIV and STI risk and HPC engagement, with the purpose of informing the development of phase 2 online survey measures and recruitment strategies. In-depth, semistructured, one-on-one interviews were conducted by trained members of the research team who also identified as members of the TNB community. Participants were purposively sampled across gender identities and racial and ethnic groups to ensure that diverse perspectives were represented. Interview participants experiencing IPV were recruited through a combination of online responses to a flyer advertising a TNB health and relationships study—posted on social media and dating sites frequently used by TNB individuals—and through referrals from TNB community health care settings. Potential participants completed a brief screener that included self-reports of recent IPV experiences (victimization or perpetration). Key informants were recruited through network referrals from community-based IPV service settings.

Interviews were conducted using secure online videoconferencing software. After orienting participants to the purpose of the interviews, answering their questions, and obtaining informed consent, the interviewer followed a semistructured protocol to guide inquiries about participants’ lived experiences of romantic relationships, experiences of different forms of violence in intimate relationships, including any forms of violence that may be specific to TNB persons, and the ways in which these experiences may directly or indirectly impact HPC engagement. For key informants, interview questions focused on their professional roles and experiences providing services to TNB individuals who had experienced IPV (victimization or perpetration), as well as on their perspectives regarding the possible impacts of IPV on HPC engagement. Interviews lasted approximately 60 minutes, and all participants were remunerated US $100 for their time.

### Phase 2 Longitudinal Cohort Data Collection

For phase 2, we plan to enroll 600 TNB individuals, 300 assigned male at birth (AMAB) and 300 assigned female at birth (AFAB), who identify as either transgender (75%) or nonbinary (25%) and report recent (past 6 months) sexual behavior with at least one person with a penis, given the study’s focus on HIV risk and HPC engagement [9]. Details on inclusion criteria and the recruitment process for phase 2 are listed below in the Recruitment section. Upon enrollment into the open prospective cohort, 600 TNB participants will be followed for 24 months. We anticipate up to 20% attrition, resulting in a final sample of ~480 TNB (240 AFAB and 240 AMAB) participants at the final 24-month assessment. This sample size was informed by Monte Carlo simulation data from Wolf et al [53], indicating that a sample of 180 provides 83% power to detect a moderate mediated indirect effect (0.4) with minimal bias; thus, enrolling 600 participants will ensure adequate power for the planned longitudinal structural equation and mediation models, allowing for robust model estimation across multiple time points, covariates, and subgroup comparisons (eg, by gender identity and AMAB or AFAB status).

**Figure 2:**
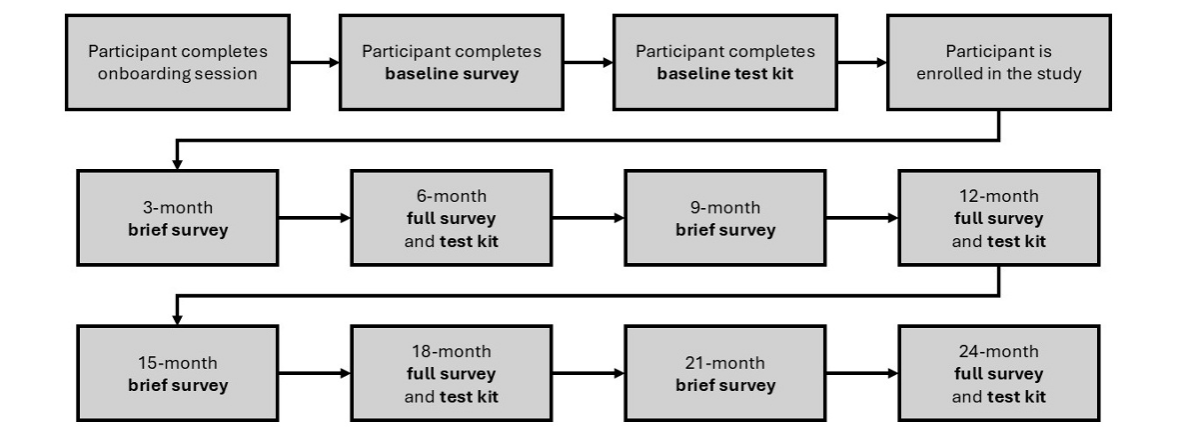
Flow diagram of the study assessment schedule in phase 2.

Full study assessments, which include completing a full-length survey and biospecimen test-kit collection, are administered at baseline and at 6-, 12-, 18-, and 24-month assessment points. At each time point, participants receive a link to an online survey that asks about their HIV and STI testing behavior, HIV status, STI infection and treatment history, and PrEP use during the past 6 months. Participants are also asked to complete a battery of demographic, psychosocial, relational, IPV, and structural measures. To maintain engagement and retention, brief interim surveys focusing on key study outcomes (eg, changes in relationship status, mental health, and HPC engagement) are administered at 3-, 9-, 15-, and 21-month intervals. The flow of the study assessment schedule is depicted in Figure 2.

All surveys and test kits are self-administered. Participants are instructed that they may contact study staff via email, SMS text message, or phone call should any questions or concerns arise. Study staff notify participants of any preliminary reactive HIV or STI test results and facilitate linkage to care within 48 hours of a reactive result. Participants receive up to 6 weekly reminders to complete their survey or return a test-kit, based on their preferred method of contact, either SMS text message or email.

### Phase 2 Recruitment

Because the primary goal of RADIANT is to understand HIV risk and HIV prevention behaviors in the context of relationship dynamics and IPV, eligibility is focused on TNB individuals who report recent sex with persons AMAB. Individuals who recently left relationships are not included in baseline eligibility, as enrolling participants in ongoing relationships is essential to prospectively capture dyadic processes and IPV-related changes in HIV prevention behaviors over the 24-month study period. As such, TNB individuals are eligible if they (1) are 18-45 years old, (2) currently identify as transgender or nonbinary, (3) report sex with a person with a penis in the past 6 months (given elevated HIV risk and the study’s focus on HIV outcomes [9]), (4) report being in a relationship for the past 3 months, and (5) have an HIV-negative or unknown status (verified at baseline via dried blood spot [DBS] assay). A detailed list of inclusion and exclusion criteria is provided in Textbox 1. We plan to stratify enrollment, as needed, to ensure that 75% of participants identify with one or more racial or ethnic minority groups, 50% are AMAB, 50% are AFAB, and at least 25% identify as nonbinary. We will also stratify to ensure that a minimum of 60% of participants report a history of IPV at baseline. This recruitment strategy will allow us to examine and compare differences in IPV experiences and their associations with HPC engagement across TNB subgroups. Based on prior work with this population, we anticipate that an additional 15% of participants who report no past-year IPV at baseline will report IPV exposure during the study, yielding a final sample of at least 400 (66.6% of study participants) with IPV exposure.

**Phase 2 study eligibility criteria.**

**Inclusion criteria**

- Currently identify as transgender, nonbinary, or another gender identity that differs from the sex they were assigned at birth
- 18-45 years of age
- Report currently being in a relationship; relationship is defined as “Do you have a primary partner, that is, someone you feel emotionally, romantically committed to above others?”
- Reside in the United States
- Have a physical (non-PO Box) address where they can receive an HIV, STI, and PrEP test kit by the US Postal Service priority mail
- Able to provide at least 2 means of contact for follow-up
- Not currently enrolled in an HIV prevention intervention study
- Have a self-reported HIV-negative serostatus at baseline (status confirmed via home-test kit mailed to laboratory)
- We may stratify eligibility as needed to ensure that at least 60% report past year IPV at baseline
- We will stratify as needed to ensure at least 35% of the sample identifies as Black or African American, and at least 35% identifies as Hispanic or Latinx
- We will stratify to ensure at least 50% were AMAB and 50% were AFAB
- We will stratify to ensure 75% identify as transgender and 25% identify as nonbinary

**Exclusion criteria**

- Under 18 years old, or older than 45 at enrollment
- Partnered for less than 3 months, or currently unpartnered
- Lives outside of the United States
- Self-reports HIV positive status or is laboratory-determined to be HIV positive at baseline
- Individual expresses unwillingness to complete regular surveys during informed consent
- Unwillingness to provide biospecimens with home testing kits during informed consent
- Unwillingness to provide partner contact information (to allow us to screen for dyads)
- Individual’s romantic partner is already enrolled in the study (we will not enroll dyads)

To ensure the participation of TNB individuals from diverse backgrounds, it has been essential to work closely with organizations and individuals within the TNB community who are connected to relevant venues and services. We continue to collaborate with members of our CAB to assess the suitability of websites, social media platforms (eg, Facebook and Instagram), and dating apps (eg, Grindr and Taimi) for online advertising of our study recruitment materials. In addition to online recruitment, we have implemented targeted strategies to reach racially and ethnically minoritized TNB individuals by partnering with community organizations to promote the study within their networks, at TNB-specific events [54], and in virtual community spaces.

**Figure 3:**
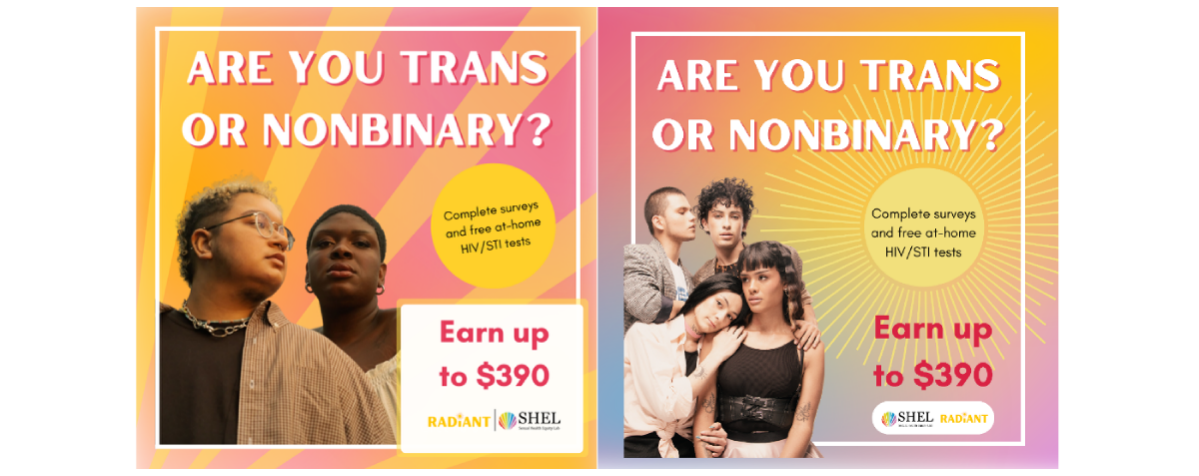
Sample phase 2 study advertisements.

Individuals interested in participating are directed from an online or offline advertisement (as shown in Figure 3) to an online study screener that asks about TNB identity and experiences of conflict and violence in intimate relationships. Drawing from methods used previously to recruit sexual and gender minorities experiencing IPV [55], we will not refer directly to IPV in recruitment efforts. Eligible participants are contacted by study staff to schedule a 20-minute virtual onboarding session to verify their identity, obtain informed consent, and provide an orientation to the study. During the consent process, participants will choose whether to receive study reminders via email or SMS text and are informed of potential privacy risks if partners access their devices. Messages will be sent from a neutral sender name and will not include any information about the study topic. Consistent with the World Health Organization ethical guidelines for IPV research [56], only one partner from any relationship will be enrolled to protect participant safety. During this onboarding session, participants are guided through the informed consent form, study assessment schedule, and biospecimen collection procedures (ie, oral and anal swabs, urine collection, and DBS collection) via written and video instructions. Once the live onboarding session has concluded, consented participants who agree to participate are sent a unique link to the baseline survey that takes approximately 1 hour to complete. Participants who complete the baseline survey are then mailed a biospecimen collection kit to test for HIV, gonorrhea, and chlamydia. For participants who reported PrEP use in their baseline survey, the kit will also include testing to assess PrEP adherence.

### Baseline Survey

During each participant’s onboarding session, the study staff complete an online enrollment form, which includes items on the participant’s name, gender identity, race and ethnicity, phone number, email, and mailing address. This information is then matched with a unique ID number (assigned consecutively by enrollment date) and a random personal identification number and securely stored in a record management system. Upon completion of the enrollment form and receipt of an electronically signed informed consent form from the participant, an automated email with a unique link to the baseline survey is sent to the participant.

The baseline survey was developed based on the findings from phase 1 interviews, past literature on IPV and HIV risk among TNB populations, and our team’s prior experience administering surveys focused on IPV and HIV prevention [55]. The baseline survey includes items centered on the following domains: experiences of IPV victimization and perpetration, including items on TNB-specific, psychological, sexual, emotional, and physical experiences of violence; HIV and STI risk and prevention, including HIV testing, PrEP uptake, adherence, and persistence; physical and mental health status and health care usage; sociodemographic and relationship characteristics; experiences of racial and transphobic stigma and discrimination; sexual behavior, particularly condomless sex; substance use; and structural and protective factors. A detailed list of proposed measures for the baseline survey is presented in Textbox 2. Some non-TNB-specific measures were modified to include TNB-inclusive language. Forsta, a survey software, was used to program the baseline survey.

Participants receive up to 3 weekly automated reminders from RAND Survey Research Group and 3 additional weekly reminders from study staff sent by participants’ preferred method of communication (SMS text or email). Participants who do not complete the baseline survey or test kit are withdrawn from the study.

**Key measures included in the baseline survey and the planned 6-month follow-up survey.**

**Intimate partner violence (IPV)**

- IPV-Transgender and Gender Diverse Populations scale (modified to be 34 items assessing IPV among transgender and gender diverse populations, including transgender and nonbinary (TNB)–specific IPV, psychological, sexual, emotional, and physical IPV) [3,33,34,57] and adapted financial control items [58], whether they consider relationship abusive, whether IPV occurred in the context of self-defense for both victimization and perpetration
- Disclosure of IPV, help-seeking behaviors, and receipt of IPV services [55,59-61]
- IPV victimization stigma and shame [62], IPV perpetration stigma and shame [63]

**HIV and STI prevention behaviors**

- HIV testing [64] (self-report and medical record confirmation)
- Sexually transmitted infection testing, diagnosis, and treatment [64] (self-report and biomarker)
- Pre-exposure prophylaxis (PrEP) uptake and PrEP persistence [64] (self-report and biomarker)
- Perceived PrEP adherence [64] (self-report)
- Reasons for not using PrEP or stopping PrEP [59-61] (self-report)
- PrEP modality acceptability [65] (self-report)
- Long-acting injectable PrEP use and acceptability [55]
- PrEP stigma [66] (self-report)
- Doxy-PEP (post-exposure prophylaxis) awareness, use, and willingness [64]
- Sexual behaviors and condomless sex (self-reported) [55,64]

**Demographics**

- Age, race, and ethnicity [67,68]
- Gender identity, sex assigned at birth, gender expression, age at which started living in true gender, intersex diagnosis or characteristics [69], sexual orientation [69]
- Educational attainment [70], employment status [70], employment precarity [71]
- Household and individual income, financial well-being [72], food insecurity [73]

**Health status and health care**

- Self-rated health [74], physical health care use [75], insurance coverage [76], bowel health [77]
- Behavioral health care use and perceived unmet need, for mental health care and substance use treatment [78]

**Partner and relationship characteristics (reported by index participant)**

- Current relationship status, marital status, cohabitation, partner demographics [55,67-69]
- Relationship characteristics (type, duration, and history of separations)
- Relationship role models, globally and specifically within TNB and the nonbinary community (regardless of relationship status)
- Relationship satisfaction [79], intimacy with partner [80], overall relationship quality and well-being [81], perceived commitment to relationship [82,83], communication patterns [84]
- Partner PrEP use or HIV treatment status or viral suppression [55,64]
- Partner knowledge of, attitudes toward, and support for taking PrEP [59-61], PrEP conversations [59-61], sexual agreements (type and adherence) [55]
- Relationship power balance and decision-making [85], financial dependence
- Social support from partner [86]

**Early life and childhood experiences**

- Adverse childhood violence and abuse, general items and items specific to sexual and gender minorities [87,88] (planned for 6-month survey), witnessed parental IPV
- Mistreatment by adults in childhood [87,89], discrimination and other minority stress experiences based on one’s gender identity or expression in childhood [51]

**Social and structural factors**

- Recent exchange or transactional sex [55,64]
- Justice system involvement (lifetime and recent) [64], experience with stop and frisk [64]
- Experienced discrimination due to race, ethnicity, or color (and frequency of these events) [90]
- Housing status and housing instability [91], recent homelessness, ever been homeless [92], perceived neighborhood safety [93]

**Mental health**

- Depressive symptoms [94], posttraumatic stress disorder symptoms [95], anxiety symptoms [96]
- Loneliness [97], social isolation symptoms [98] (planned for 6-month survey)
- DSM-5 (*Diagnostic and Statistical Manual of Mental Disorders* [Fifth Edition]) cross-cutting symptoms [99] (planned for 6-month survey)
- Sleep quality [100] (planned for 6-month survey)
- Emotional regulation ability [101] (planned for 6-month survey)

**Substance use and abuse**

- Alcohol use [102], illicit and licit substance use [103], substance use consequences [103]

**Psychosocial and resilience factors**

- Internalized societal gender roles [104], comfort with gender identity [105]
- Discrimination and other minority stress experiences due to gender identity or expression in adulthood, past year [51], anticipated stigma (global demographics) [106]
- Discrimination due to sexual orientation, frequency [107] (planned for 6-month survey)
- Connectedness to TNB and nonbinary community [108]
- Perceived social support (global) (eg, emotional and instrumental) [86]
- Coping self-efficacy [109], global resiliency traits [110,111], global self-esteem [112] (planned for 6-month survey)

### Biospecimen Sample Collection Procedures

Upon completion of the baseline survey, participants are mailed a biospecimen collection kit by the study’s designated lab partner, using the address provided during onboarding. Kits are shipped via the US Postal Service in plain, discreet packaging and include a prepaid return label. Each kit contains DBS cards, a urine collection cup, oral and anal swabs, collection tubes, lancets, and detailed self-collection instructions. Participants begin receiving automated weekly reminders via SMS text or email 1 week after the kit is mailed, up to 6 reminders in total. Participants who do not return their kit after 6 reminders and wish to remain in the study will be encouraged to complete a new kit. Those who do not return a kit or test positive for HIV at baseline will have their baseline survey data retained but will be withdrawn from the cohort and excluded from further study activities.

Biospecimen kits are also mailed at baseline, 6-, 12-, 18-, and 24-month follow-ups, with contents tailored based on the study time point and the participant’s PrEP use. DBS samples are collected to measure PrEP adherence (when use is self-reported) at baseline, 6, 12, 18, and 24 months. HIV is measured at baseline (to determine eligibility) and again at 24 months. For STI testing (chlamydia and gonorrhea), participants self-collect urine (30-50 mL from the initial stream), as well as rectal and pharyngeal swabs at baseline, 6, 12, 18, and 24 months. Urine samples are tested via nucleic acid probe, and swabs are analyzed using nucleic acid amplification. Table 1 presents the full schedule of survey assessments and biospecimen collection activities.

**Table 1:** Schedule of survey assessment and biospecimen collection by time point^a^.

Outcome			Study time point								
			Baseline		6-month		12-month		18-month		24-month
**Primary**											
	HIV testing behavior	Survey		Survey		Survey		Survey		Survey	
	PrEP^b^ uptake	Survey + DBS^c^		Survey + DBS		Survey + DBS		Survey + DBS		Survey + DBS	
	PrEP persistence	Survey + DBS		Survey + DBS		Survey + DBS		Survey + DBS		Survey + DBS	
	STI^d^ (CT^e^ and GC^f^)	Survey + culture		Survey		Survey + culture		Survey		Survey + culture	
**Secondary**											
	Sexual risk behavior	Survey		Survey		Survey		Survey		Survey	
	HIV seroconversion	Survey + DBS		Survey		Survey		Survey		Survey + DBS	

^a^Brief assessments of relationship changes, experiences of intimate partner violence, and self-reported HIV prevention continuum and STI outcomes will be administered at 3, 9, 15, and 21 months (not shown).

^b^PrEP: preexposure prophylaxis.

^c^DBS: dried blood spot.

^d^STI: sexually transmitted infection.

^e^CT: chlamydia.

^f^GC: gonorrhea.

### Laboratory Testing and Follow-Up

Biospecimen testing is conducted by our external lab partner. If a sample is determined to be insufficient, participants are contacted via their preferred communication method (email or SMS text) to request a second collection. A replacement kit is mailed by the lab, and participants are asked to recollect and return the sample by mail. Test results are shared with study staff through the lab’s secure online portal. If a result is reactive, study staff contact the participant by phone to deliver the result verbally, confirm their identity, and explain that the testing was conducted for research purposes only. Participants will be encouraged to seek confirmatory testing from a medical provider and offered referrals to local resources. Upon request, study staff will provide an electronic copy of the test results, which will be password-protected to ensure secure transmission of protected health information.

### Study Communication and Participant Retention

The study uses multiple methods of communication to maintain participant database, program and send automated study task reminders, and contact participants to follow up on unfinished study activities and reactive test results. Participant information, survey response data, test results, and other administrative data are stored and maintained separately, each in securely encrypted online databases. Most contacts with the participants will be made via SMS text messages and emails, in the forms of automated messages or prewritten templates sent by the study staff. These messages will use conversational tones and an accessible reading level. Videoconferencing will be used for the initial onboarding sessions, with follow-up sessions made available on request regarding test kit completion.

### Compensation

Participants receive US $20 for completing each full-length survey at baseline, 6-, 12-, 18-, and 24-month follow-ups, and US $40 for returning each corresponding test kit with sufficient biospecimen for analysis. They will also receive US $10 for each brief survey completed at 3, 9, 15, and 21 months, which assess relationship status changes, HPC engagement, and STI diagnoses and treatment. Participants who complete all 5 full-length surveys and return all 5 corresponding test kits will receive a US $50 bonus. In total, participants may earn up to US $390 for full study participation. All compensation is provided as electronic gift cards.

### Data Analysis Plan

#### Overview

This study uses both gender-inclusive and gender-specific approaches to analyze experiences of IPV and HPC engagement across all phases of the research. This analytic framing is aligned with current guidance for research involving TNB populations [113] and is supported by evidence that transfeminine, transmasculine, and nonbinary individuals often report distinct experiences of violence [108,109], as well as differing barriers and facilitators to HPC engagement [23]. Using both gender-inclusive and gender-specific approaches is necessary to identify shared as well as unique patterns across groups.

In phase 1, qualitative data are being examined both across all participants and separately within transfeminine, transmasculine, and nonbinary groups to explore common themes and preserve distinct narratives and lived experiences. In phase 2, quantitative analyses will include longitudinal modeling to assess changes in IPV and HIV prevention outcomes over time, using both full-sample and subgroup-specific models. This combined approach will allow the study to generate evidence that supports both broadly applicable recommendations for all TNB participants and targeted insights that address the specific needs of transfeminine, transmasculine, and nonbinary individuals. These findings will contribute to the development of more effective and culturally responsive interventions to address IPV and support HIV prevention.

The following section outlines the analytic approaches for data collected in phases 1 and 2 of the study. While the central focus will remain on examining IPV experiences and their associations with HPC engagement and STI outcomes, specific analytic methods may be refined based on the characteristics of the data (eg, distributional properties), emerging research questions, and input from the study statistician. Additional analyses will also be conducted to explore secondary outcomes of interest as appropriate.

#### Phase 1 Qualitative Data Analyses

To analyze the qualitative interview data from phase 1, grounded theory was used to allow themes to emerge from the data [114]. Interviews were audio-recorded and transcribed verbatim and reviewed independently by investigators to identify analytic thematic categories that emerged in response to the interview topics. The transcripts were reviewed periodically to determine whether thematic saturation had occurred, using a saturation grid [115]. Additional interviews were conducted until saturation was achieved. Investigators independently developed an initial list of themes and then developed a codebook listing each theme accompanied by a detailed description, inclusion and exclusion criteria, and typical examples. Dedoose (version 7; Sociocultural Research Consultants, LLC) was used for coding. Two coders marked areas of text pertaining to each theme. They practiced with a sample of 20% of transcript selections, coding independently and reviewing together. If coder disagreement reveals ambiguity in the codebook, code definitions, examples, or criteria are revised as needed. Training continues until coders consistently identify themes.

Next, both coders work on each passage independently, after which the research team measures coder consistency, evidenced by a weighted kappa of ≥0.7, a more rigorous approach than simple percent agreement [116]. Best practices for validity are used, including triangulation and an audit trail [117]. Distribution of themes within and across age, gender, racial and ethnic identity, IPV type, frequency, severity, and community member versus provider status are examined to determine whether there are differences in perceptions of associations of various forms of IPV and HIV risk and HPC engagement.

Interview findings were used to help refine measures to be used in phase 2 in the cohort study by building upon the research team’s preliminary work with community partners and experts on IPV among TNB persons. The themes that emerged from phase 1 interviews helped to contextualize the knowledge on TNB-specific forms of IPV provided by participants and key informants and consequently informed survey development in phase 2, like selecting relevant measures and developing relevant items. Qualitative and quantitative data will be brought together again at the end of the quantitative analysis phase to assess complementarity [118]. An overall summary of study findings that includes the most salient aspects of IPV in relation to HIV risk and HPC engagement gleaned from the quantitative analyses with complementary qualitative data will be developed.

#### Phase 2 Quantitative Data Analyses

In phase 2, we will examine the robustness of our measures and our sample, with particular attention to participant attrition and patterns of missing data. First, we will assess the psychometric properties of all measures. Second, we will perform Wilcoxon and chi-square tests to compare baseline and follow-up characteristics between participants who completed the study and those who did not. Statistical methods will be applied to adjust for potential bias due to attrition [119]. To address missing data, we may use standard multiple imputation approaches [120], including the use of sequential Bayesian additive regression trees (R package “sbart”), a nonparametric method that does not rely on assumptions about covariate relationships [121]. Before building more elaborate latent curve models (LCMs), we will conduct preliminary analyses—such as bivariate correlations, regression models, and basic structural equation modeling (SEM) and LCMs—to examine associations among key study variables.

To evaluate the proposed study aims with 5 waves of full data collection (baseline, 6, 12, 18, and 24 months), we will apply SEM and specifically LCMs to examine the trajectories of one or more outcome variables over time. LCM is a flexible technique for modeling systematic within- and between-individual differences in longitudinal change and offers several well-documented advantages over other methods [122,123]. LCM will be used to model multiple parallel developmental trajectories of change and the relations between them (eg, between the predictor IPV measures and the outcome HPC measures). Another advantage of LCM is that one can incorporate multiple indicators to form a “measurement model” that teases out the measurement error from observed behaviors [124]. LCM allows for testing complex relationships between the predictor and outcomes with time-invariant and time-varying covariates. We will also use longitudinal latent class analysis [125,126] to identify phenotypes that may extend beyond groups of gender identity, for example, family history, substance use, incarceration, IPV subtypes, and geographic differences, therefore enabling us to understand relationships between IPV and HIV outcomes and identify groups that may benefit from tailored interventions.

One of the aims of the study is to examine gender-based differences in the longitudinal associations of IPV with HPC engagement, STI diagnosis, condomless sex, and HIV seroconversion among a racially, ethnically, and gender diverse cohort of TNB persons. To address this aim, we will model multiple developmental trajectories of 5 full survey (baseline, 6, 12, 18, and 24 months) waves of data using LCM for transgender women, transgender men, and nonbinary subgroups. There are three developmental trajectories we will examine for each group: (1) the predictor: IPV; (2) the primary outcomes: HPC engagement and STI diagnoses; and (3) the secondary outcomes: condomless sex and seroconversion. The predictor trajectory is defined by repeated measures of IPV. Because LCM enables the simultaneous estimation of multiple developmental pathways, we will model parallel trajectories of HPC engagement across subgroups. Key outcomes will be derived from repeated measures of 3 binary indicators: HIV testing, PrEP initiation, and PrEP continuation. These indicators will be used to define a single latent HPC factor (*f*). With the 5 full survey waves of data, the repeated measures of the same latent variable are represented by *f*1 to *f*5 in the LCM. The developmental trajectory of HPC engagement will then be based on the latent variables *f*1 to *f*5. Similarly, we will examine parallel developmental trends in outcomes such as STI diagnoses, condomless sex, and HIV seroconversion. To assess the shape of these trajectories, we will evaluate whether growth is best represented as linear or nonlinear, incorporating quadratic terms or piecewise models if needed. Further, we will have multiple parallel developmental processes (eg, IPV, HPC, and STIs) in the growth model.

The study also aims to determine the individual-, interpersonal-, network-, and structural-level risk and resilience factors that mediate (or moderate) the associations between IPV and HIV risk and protective behaviors for each group. It is hypothesized that resilience factors, such as coping skills, greater social support, and positive role models, will act as a mediator or a moderator in the relationships that IPV will have with HIV risk and HPC outcomes. We also hypothesize mediating effects of potential risk factors (eg, substance use, poorer mental health, engagement in transactional sex, incarceration, and partner- and relationship-level factors). Such potential mediating effects will be incorporated into SEM.

Because of the complexity of the LCM approach with multiple developmental trajectories, we anticipate challenges in adding moderators directly into the growth model. One way to address this is by using multiple group analyses as a strategy for evaluating moderation effects across subgroups. By comparing relationships between the predictor trajectory and the outcome trajectories across different groups (transfeminine, transmasculine, and nonbinary individuals or by groups identified in the LCA), the multiple group analyses will allow us to test different assumptions about group equality [127] and build appropriate models for different, heterogeneous subpopulations. In addition, by incorporating measures of resilience, this study supports the design of strength-based interventions, building on evidence that resilience can serve a protective, buffering role [128].

We will incorporate both time-varying and time-invariant covariates into the LCMs to examine the influence of individual characteristics of TNB participants, their partners, and relationship dynamics. Analyses will be conducted using Mplus Version 7 (Muthén & Muthén). We will examine a range of moderation and mediation effects to assess how various risk and protective factors influence outcomes, accounting for demographic and socioeconomic variables. Alternative models will be compared using a set of model fit indices, including root-mean-square error of approximation, Tucker-Lewis index, and various fit statistics as described by Bentler and Bonett [129], and Hu and Bentler [130].

### Ethical Considerations

All study protocols and procedures have been approved by the San Diego State University institutional review board (HS-2023-0142) on July 28, 2023. All procedures are in accordance with the ethical standards of the institutional and national research committees and with the Helsinki declaration and its later amendments or comparable ethical standards. Verbal and written informed consent have been and will be obtained from all participants included in the study following a thorough individual study onboarding process carried out by research team members, conducted via online videoconferencing software. Participants are informed that their involvement in the study is voluntary and that they may withdraw at any time without penalty. Certain personal details (eg, name, mailing address, postal code, email, and phone number) are collected for study administration. To maintain privacy, only authorized research personnel trained in data protection and confidentiality have access to identifiable information. Electronic files are stored on secure, encrypted servers with restricted access, and identifiers are kept separate from research data. All data transfers are encrypted, and data will be permanently deleted once they are no longer required for research purposes. Confidentiality protections are explained during the informed consent process.

## Results

This study was funded in September 2023 by the National Institute of Mental Health (R01MH133484; to EDS). The RADIANT study was launched in October 2023, and phase 1 interviews were initiated in April 2024 and were conducted through October 2024 until thematic saturation was reached. Rapid qualitative analysis was conducted between November 2024 and January 2025 to inform phase 2 survey programming. Formal analysis of the qualitative data is currently ongoing, and findings will be submitted for peer-reviewed publication. The phase 2 baseline survey was finalized in January 2025, and recruitment for phase 2 began in early February 2025. Enrollment is expected to continue through December 2025. Phase 2 cohort participants are expected to complete all follow-up assessments by December 2027.

## Discussion

### Principal Findings

This will be the first longitudinal prospective study of IPV and HPC engagement for gender diverse populations, allowing us to better understand potential mechanisms between IPV and HIV risk and protective factors. We will assess multiple forms of IPV, including TNB-specific (eg, controlling gender expression), psychological, sexual, emotional, and physical, unlike most studies that have focused on physical and sexual forms of abuse. Broader research on IPV suggests that psychological and emotional abuse also have significant impacts [56,131-134]. Yet, these forms of abuse and their effects are understudied among TNB persons [2,3,33]. Our approach to measuring IPV includes both victimization and perpetration, helping to address a significant gap in the existing literature, which has often overlooked IPV perpetration among TNB individuals and focused primarily on their experiences as victims [2]. Additionally, our study introduces a novel focus on the chronicity of IPV, as well as partner characteristics and relationship contexts in which violence occurs. Unlike most prior research, which typically assesses IPV over broad time frames such as lifetime or past year, our design allows for the examination of patterns over time—such as repeated episodes, changes in intensity, and the progression or reduction of violence.

In taking a multidimensional, longitudinal assessment of TNB-specific IPV and HPC outcomes among TNB persons, this study will be able to examine how the associations between IPV and HPC vary for transfeminine, transmasculine, and nonbinary individuals. We will assess the interaction of multiple syndemic factors among specific gender identity groups. We build on a methodological design that we have refined over several previous studies, including a similar longitudinal IPV study with sexual minority men [55]. This will allow us to explore the unique and common effects of different kinds of stigmas and supportive factors on HIV risk and HIV prevention outcomes pertaining to the TNB population.

A longitudinal approach allows us to assess the temporality of associations between IPV and HPC engagement and heterogeneous phenotypes therein. This study will allow us to distinguish between TNB individuals who experience IPV concurrently with low engagement in the HPC and those whose IPV precedes declines in HPC engagement or increased HIV risk behaviors. We will also be able to evaluate whether greater engagement in affirming, comprehensive HPC services is associated with reductions in IPV over time [135]. The longitudinal design provides the opportunity to track changes in potential mediators and moderators—such as gender identity, mental health conditions, psychosocial stressors, social support, and resilience factors—across multiple time points. While a longitudinal approach is essential for capturing these complex dynamics, it also requires a sufficiently large and diverse sample, along with adequate follow-up duration, to meaningfully examine how IPV and HIV risk evolve across different TNB subgroups and relationship types—goals that are central to this study.

Another facet of the research will focus on resiliency and protective factors for IPV and HIV prevention within the lived experiences of TNB persons. Resiliency factors such as coping skills and social support have been linked to reduced HIV-risk behavior and increased HIV testing and PrEP use [15,136,137]. Informed by both of our qualitative and quantitative data, we expect to build upon existing research by examining potential protective roles of resilience at the individual level, such as coping skills [110,138-140], social support from within one’s social network [141-144], positive self-esteem [145,146], stable employment [147-149], spirituality [150-152], adaptive coping skills [153,154], and emotional regulation [145,155,156], and at the community level, such as TNB role models and TNB-specific support networks [157-159], in buffering against the magnitude of stress- and trauma-related harm resulting from IPV. Many facets of resiliency are modifiable; therefore, understanding how resilience, coping skills, social network characteristics, and social support serve to buffer against IPV among TNB is imperative to developing culturally appropriate and strength-based interventions.

### Limitations and Strengths

This study has several limitations that are important to acknowledge. First, data on both IPV victimization and perpetration will be based on self-reports, which may introduce bias. Perpetration may be underreported due to concerns about potential legal implications, while victimization could be underreported as a result of social desirability or stigma. To help address these challenges, in-depth qualitative interviews will be used to gain insight into how best to assess both victimization and perpetration in the context of romantic relationships among TNB persons. The prospective design of the study also enables us to assess how current IPV experiences influence downstream outcomes related to HPC engagement. Second, our assessment of perceived social support, a potential buffering factor in the relationship between IPV and HPC-related outcomes, is based on an egocentric measure that captures individuals’ perceptions of support from various members of their social networks (eg, peers, family, and coworkers). While this approach may have limitations, egocentric data collection using validated tools is a well-established and widely accepted methodology in research.

Despite these limitations, this study is grounded in a rigorous methodological approach and has strong potential for public health impact. Our interdisciplinary team brings extensive expertise in prospective cohort study design, IPV research, HIV prevention among sexual and gender minority populations, and advanced statistical modeling. We will collect data on exposures, moderators, and outcomes at multiple time points, allowing for a comprehensive analysis of dynamic relationships over time. Importantly, this study will yield actionable findings to inform the development of targeted interventions aimed at reducing both IPV and gaps in HPC engagement among TNB persons. To our knowledge, this will be the first study of its kind to produce the scientific evidence necessary to guide intervention strategies that address these intersecting health risks, aligning with priorities outlined in the National Institutes of Health Strategic Plan for HIV and HIV-Related Research.

### Conclusions

The RADIANT study will be designed and implemented with a high degree of scientific rigor and has the potential for greatly increasing the understanding of the pathways by which specific forms of IPV have direct and indirect effects on HIV- and STI-related outcomes. Through the development of actionable recommendations for intervention design, this study is positioned to be the first to generate the foundational evidence needed to guide effective strategies aimed at mitigating the dual harms of IPV and HIV among TNB individuals. These findings will address a critical gap in the field and contribute meaningfully to national efforts to end the HIV epidemic.

## Abbreviations

AFAB: assigned female at birth
AMAB: assigned male at birth
CAB: community advisory board
DBS: dried blood spot
HPC: HIV prevention continuum
IPV: intimate partner violence
LCM: latent curve model
PrEP: pre-exposure prophylaxis
RADIANT: Relationships And Dynamics—Improving Advocacy for Nonbinary and Trans
SEM: structural equation modeling
STI: sexually transmitted infection
TNB: transgender and nonbinary
